# Carotid Web Diagnosed by Ultrasound Carotid Duplex in a Patient With Ischemic Stroke

**DOI:** 10.7759/cureus.16330

**Published:** 2021-07-12

**Authors:** Pilar Calle La Rosa, Rosa Ecos, Ricardo D Otiniano-Sifuentes, Jorge Ramírez-Quiñones, Carlos Abanto, Darko Quispe-Orozco, Ana Valencia

**Affiliations:** 1 Neurovascular Diseases, National Institute of Neurological Sciences, Lima, PER; 2 Neurology, University of Iowa Hospitals and Clinics, Iowa, USA

**Keywords:** carotid web, fibromuscular dysplasia, ischemic stroke, carotid bulb, ultrasound carotid duplex

## Abstract

Carotid web (CW) is an atypical form of intimal fibromuscular dysplasia that occurs at the level of the carotid bulb. It is associated with ischemic strokes. The first report of this association was in 1967 and it is currently known to represent a significant percentage of cryptogenic stroke. We report the case of a young female patient with a history of transient ischemic attack who presented a cerebral infarction of the territory of the left middle cerebral artery. The diagnosis of CW was suggested by the findings of the ultrasound carotid duplex and was confirmed by digital subtraction angiography. Likewise, brain magnetic resonance angiography showed an incipient alteration in the morphology of the wall of the left internal carotid artery in its intracranial segment. Aspirin treatment was started and there was no recurrence up to two years of follow-up. CW represents a diagnostic challenge; it should be suspected in young adults with ischemic stroke. In them, studies of the supra-aortic vessels should be performed. Ultrasound carotid duplex can be a useful diagnostic tool.

## Introduction

Carotid web (CW) is an atypical form of intimal fibromuscular dysplasia (FMD) that occurs at the level of the carotid bulb. It is due to abnormal fibrosis and hyperplasia of the intimal layer [1]. Its diagnosis is based on the angiographic study [1,2]. CW is associated with recurrent ipsilateral ischemic stroke. The first report of this association with ischemic stroke was in 1967 by Ehrenfeld et al. and it is currently known to represent a significant percentage of cryptogenic ischemic stroke [3].

We describe the case of a patient who had a transient ischemic attack (TIA) and ischemic stroke, due to CW, diagnosed by carotid duplex ultrasound (UCD). Incipient findings of involvement at the level of the intracranial artery wall were also found. This article was previously presented as a meeting abstract at the 2021 Virtual Annual Meeting of the American Academy of Neurology.

## Case presentation

We present the case of a 35-year-old woman, with no history of cardiovascular risk factors or drug abuse, but with the antecedent of spontaneous abortion in the first trimester of pregnancy, three years before the current event and without any complications. Four months before her admission, she presented an episode of TIA characterized by right brachial monoparesis and expression aphasia, which lasted approximately 30 seconds with total remission of symptoms.

**Figure 1:**
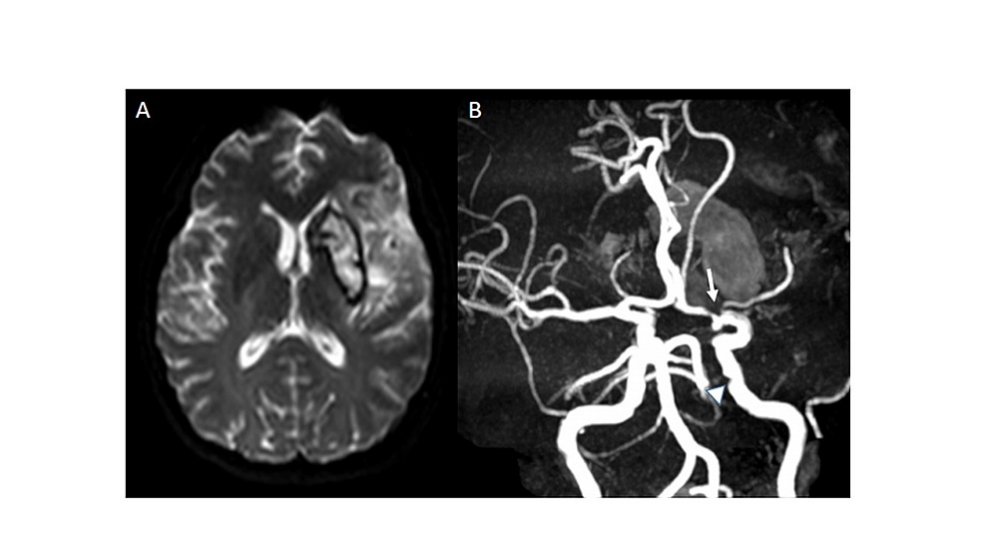
Acute ischemic stroke (A) Brain MRI, apparent diffusion coefficient map protocol shows subacute cerebral infarction in the territory of the left MCA. (B) MRA shows an occlusion in the M1 segment of the left MCA (arrow). In addition, there is evidence of alteration in the morphology of the intracranial wall of the ICA ipsilateral to the lesion (arrowhead). MRI, magnetic resonance imaging; MCA, middle cerebral artery; MRA, magnetic resonance angiography; ICA, internal carotid artery.

**Figure 2:**
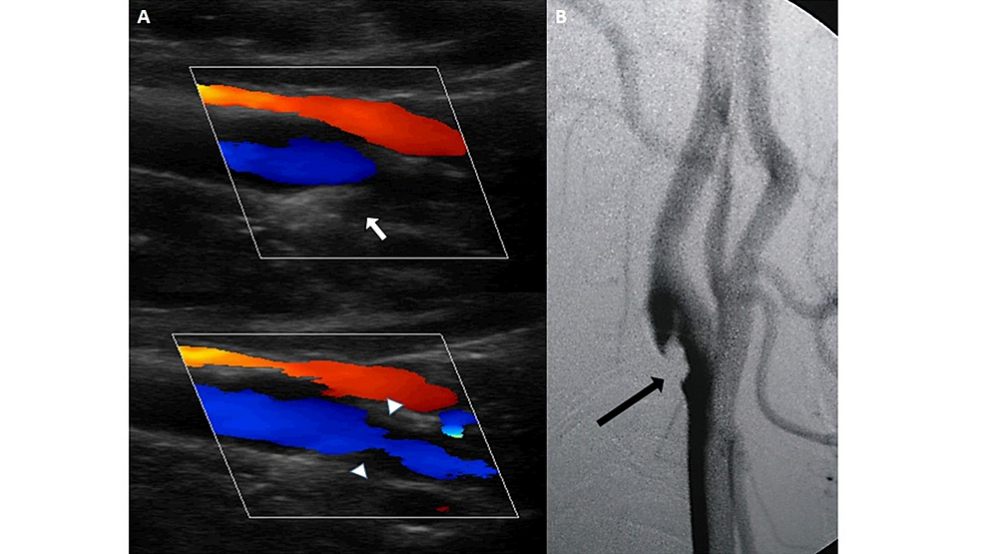
Carotid web (A) Ultrasound carotid duplex shows intimal thickening greater than 0.9 mm and the presence of an isoechoic membrane of 0.75 cm x 0.25 cm at the level of the left internal carotid artery that produces 50% narrowing (arrow) and turbulent flow (arrowhead) with increased systolic velocity of 137 m/s. (B) In digital subtraction angiography, a shelf-shaped filling defect is observed at the level of the posterior wall of the left carotid bulb (arrow) that extends to its ascending cervical portion. (C1).

The patient was admitted to our institution for weakness in both right limbs and a sudden-onset language disorder. The neurological examination revealed right hemiparesis, right hemihypoesthesia, and global aphasia, with a National Institutes of Health Stroke Scale at the admission of 13 points. Magnetic resonance imaging showed ischemic stroke with hemorrhagic transformation in the territory of the left middle cerebral artery (MCA) (Figure 1A). Furthermore, brain magnetic resonance angiography (MRA) showed an occlusion in the proximal left MCA and alteration of the morphology of the internal carotid artery (ICA) wall in its intracranial segment (Figure 1B). The UCD evidenced the presence of a 0.75 cm x 0.25 cm isoechoic membrane at the level of the left ICA bulb with turbulent flow and increased systolic velocity (137 cm/s) (Figure 2A). Moreover, the digital subtraction angiography (DSA) displayed a shelf-shaped filling defect in the posterior wall of the left carotid bulb (Figure 2B). The transthoracic echocardiogram and the 24- and 48-hour Holter monitoring were normal. The auxiliary tests showed negative results for hypercoagulable states. Aspirin treatment (100 mg/day) was started, with a favorable clinical evolution and no recurrence until two years of follow-up.

## Discussion

We report the case of a young patient with cerebral ischemic events in the territory of the left ICA. In the UCD, findings compatible with CW were found. This entity was described for the first time in 1968 by Rainer et al. in a 30-year-old patient with TIAs [4], although it was Momose and New who in 1973 used the term “web-like tissue” for the first time to describe an intraluminal protrusion in the cervical portion of the ICA [5]. Histologically, CW presents a focal fibrotic intima dependent on the posterior wall of the carotid bulb with a shelf-like projection to the lumen with no typical atherosclerosis change [2]. This, unlike the classic form of intimal FMD, is characterized by a collagen deposition within the intima with the compromise of the internal elastic lamina, duplicating or fragmenting it, causing a focal fibrotic constriction in the form of a concentric band [6]. Both cases differ from an atherosclerotic plaque, which typically presents a large nucleus lipid-rich necrotic covered by a fibrous cap [2].

CW represents between 9.4% and 37% of ischemic strokes of undetermined etiology [3]. It has been proposed as a mechanism of the infarction that this carotid membrane produces turbulent flow and arterial stasis that promotes a thrombogenic environment. This would favor the formation of thrombi in situ and the risk of arterio-arterial embolism toward the distal branches of the anterior cerebral circulation. This is based on the angiographic images demonstrating marked stasis of contrast at the level of the carotid membrane [2,7,8]. Our patient had two ischemic events of the anterior circulation ipsilateral to the CW; also, the MRA shows an occlusion at the level of the MCA compatible with an embolic mechanism (Figure 1B).

Our case fits the epidemiological characteristics of symptomatic CW since it occurs more frequently in young women, with an average age of 46 years, without risk factors for cerebrovascular disease [2,3,7]. Choi et al. reported an angiographic prevalence of 1.2% in 576 patients diagnosed with acute ischemic stroke [2]. Compagne et al. found that this prevalence is higher (2.5%) among patients with ischemic stroke due to occlusion of a major intracranial artery [9]. Most of the described cases are from North America and Europe [8]. According to our bibliographic review, this case represents the first reported in Peru.

As part of the etiological search for ischemic stroke in our case, a UCD was performed that revealed a membrane at the level of the posterior wall of the bulbar carotid. This typical feature of CW can be appreciated by ultrasound images because the ICA is superficial and the membrane is located exclusively on the posterior wall of the carotid bulb [10]. But the knowledge of this entity is required by the person who performs the procedure. There are ultrasound findings suggestive of CW as a band of tissue that projects toward the lumen of the vessel producing a focal narrowing of the lumen, a high-flux jet, and an aliasing effect on the membrane seen on color Doppler. Also, there should be no findings consistent with atherosclerotic plaque. However, even so, its detection is often difficult and in the literature there are few reports with descriptions of UCD [10]. The diagnosis was confirmed with DSA (Figure 2B), based on the angiographic appearance of a shelf-like shaped filling defect at the level of the bulbar carotid dependent on its posterior wall [1]. This finding is best seen on an oblique sagittal angiographic image, while on the axial image it is seen as a septum dividing the artery [2].

An important finding was the evidence of an incipient filling defect at the level of the left intracranial ICA, which distorted the morphology of its wall and did not have the characteristics of an atherosclerotic plaque (Figure 1B). However, it cannot be conclusively stated that it is a membrane. There are no reports of membranes at the intracranial level and even the simultaneous involvement of the other types of FMD with CW is rare; however, in FMD patients, multiple artery involvement is documented in approximately two-thirds of cases [6]. Given this high percentage of coexistence in patients with FMD, the search for involvement at another level, especially intracranial, must be carried out.

Our patient had a TIA event and then an ischemic stroke, reflecting the high recurrence of thromboembolic events in CW [7]. However, the most appropriate secondary prevention treatment is not defined. Of the reported cases, the most frequently used medical treatment is antiplatelet drugs, 91% [7]. However, Joux et al. report a recurrence rate of 30% in a group of 20 Afro-Caribbean patients treated with antiplatelets [3] and Zhang et al. in a systematic review indicate a 54% recurrence rate, both with a median time to the new event of 12 months [7]. In theory, anticoagulants would be more effective due to a better outcome in low-flow states with significant stasis; however, in a recent systematic review, the recurrence rate was high in patients treated with anticoagulants, although the sample in this group was low (N = 3/4, 75%). In contrast, no recurrence was reported in cases treated with endovascular stenting or carotid endarterectomy, although there were losses to follow-up [7]. Despite the evidence in favor of carotid revascularization, our patient had no recurrence in two years of follow-up with antiplatelet treatment.

## Conclusions

CW represents a diagnostic challenge and should be suspected mainly in young adults with ischemic stroke. In them it is important to carry out studies of the supra-aortic vessels. UCD is a safe and low-cost alternative to detect findings suggestive of CW. Early detection is important due to its high embolic risk.

## References

[1] Kliewer MA, Carroll BA (1991). Ultrasound case of the day. Internal carotid artery web (atypical fibromuscular dysplasia). Radiographics.

[2] Choi PM, Singh D, Trivedi A (2015). Carotid webs and recurrent ischemic strokes in the era of CT angiography. AJNR Am J Neuroradiol.

[3] Joux J, Chausson N, Jeannin S (2014). Carotid-bulb atypical fibromuscular dysplasia in young Afro-Caribbean patients with stroke. Stroke.

[4] Rainer WG, Cramer GG, Newby JP, Clarke JP (1968). Fibromuscular hyperplasia of the carotid artery causing positional cerebral ischemia. Ann Surg.

[5] Momose KJ, New PF (1973). Non-atheromatous stenosis and occlusion of the internal carotid artery and its main branches. Am J Roentgenol Radium Ther Nucl Med.

[6] Olin JW, Sealove BA (2011). Diagnosis, management, and future developments of fibromuscular dysplasia. J Vasc Surg.

[7] Zhang AJ, Dhruv P, Choi P (2018). A systematic literature review of patients with carotid web and acute ischemic stroke. Stroke.

[8] Elmokadem AH, Ansari SA, Sangha R, Prabhakaran S, Shaibani A, Hurley MC (2016). Neurointerventional management of carotid webs associated with recurrent and acute cerebral ischemic syndromes. Interv Neuroradiol.

[9] Compagne KCJ, van Es ACGM, Berkhemer OA (2018). Prevalence of carotid web in patients with acute intracranial stroke due to intracranial large vessel occlusion. Radiology.

[10] Luo X, Li Z (2019). Ultrasonic risk stratification of carotid web. Echocardiography.

